# Bucal Mucosa Graft in Long Anterior Urethral Stenosis – Dorsal or Ventral?

**DOI:** 10.1590/S1677-5538.IBJU.2016.03.01

**Published:** 2016

**Authors:** Luciano A. Favorito

**Affiliations:** Professor Associado da Unidade de Pesquisa Urogenital da Universidade do Estado de Rio de Janeiro.; Urologista do Hospital da Lagoa Federal, Rio de Janeiro. Editor Associado da International Braz J Urol

The May-June 2016 issue of the International Braz J Urol presents original contributions with a lot of interesting papers in different fields: Urinary Incontinence, Urethral Stricture, Bladder Cancer, Pelvic-Ureteric Junction Stenosis, BPH, Prostate Cancer, Renal stones, Uroginecology, Pediatric Urology and basic research. The papers come from many different countries such as Brazil, USA, Turkey, Italy, Austria, Australia, Israel, Netherlands, India, Mexico, China, Saudi Arabia, United Kingdon, Korea and France, and as usual the editor ´s comment highlights some papers. We decided to comment 2 papers about a very usual topic in urologic practice: The Urethral Stricture.

Doctor Prabha and collegues from India performed on page 564 an interesting study about the single stage dorsolateral onlay buccal mucosal urethroplasty for long anterior urethral strictures. The authors studied 20 patients with urethral strictures: Lichen sclerosis in 12 cases (60%), Instrumentation in 5 cases (25%), and unknown in 3 cases (15%). Strictures were approached through a perineal skin incision and penis was invaginated into it to access the entire urethra. All the grafts were placed dorsolaterally, preserving the bulbospongiosus muscle, central tendon of perineum and one-sided attachement of corpus spongiosum. The mean stricture length was 8.5cm (range 4 to 12cm) and the overall success rate was 85%. There were 3 failures (meatal stenosis in 1, proximal stricture in 1 and whole length recurrent stricture in 1). Other complications included wound infection, urethrocutaneous fistula, brownish discharge per urethra and scrotal edema. The authors concluded that dorsolateral buccal mucosal urethroplasty for long anterior urethral strictures using a single perineal incision is simple, safe and easily reproducible by urologists with a good outcome.

The success of urethroplasty using bucal mucosa graft (BMG) is significantly better compared to others grafts (1). The BMG placement can be ventral, dorsal and lateral, but the first 2 are most commonly done (2). Dorsal placement of the graft has the advantage of using the corporal bodies to provide a secure well-vascularized graft bed that helps to prevent the protrusion of the graft with resulting pseudodiverticulum formation (3). Ventral location provides the advantages of ease of exposure and good vascular supply by avoiding circumferential rotation of the urethra (4). Early success rates of dorsal and ventral onlay with BMG were 96 and 85%, respectively. However, long-term follow-up revealed essentially no difference in success rates (5-8). Most recently, in a interestig meta-analysis review of the literature on dorsal or ventral graft urethroplasty the success rates of ventral onlay urethroplasty (750 cases) and dorsal onlay (513 cases) were 82.5 and 86.9% (p = 0.034) (9).

We can conlude that the two techniques (Ventral and Dorsal BMG) had similar sucess results in long anterior urethral strictures and the surgeon experience and preference with the technique is the most important factor for the sucess of the surgery.

Luciano A. Favorito, MD, PhD Professor Associado da Unidade de Pesquisa Urogenital da Universidade do Estado de Rio de Janeiro Urologista do Hospital da Lagoa Federal, Rio de Janeiro Editor Associado da International Braz J Urol
